# Supplementary material for: Host transcriptional responses to gut microbiome variation arising from urbanism
Source: bioRxiv. 2025 Oct 26:2025.10.26.683539. Preprint. [Version 1] doi: 10.1101/2025.10.26.683539 (PMC12633242; doi:10.1101/2025.10.26.683539)
Supplement: 1 [file NIHPP2025.10.26.683539V1-supplement-1.pdf]

SUPPLEMENTARY FIGURES

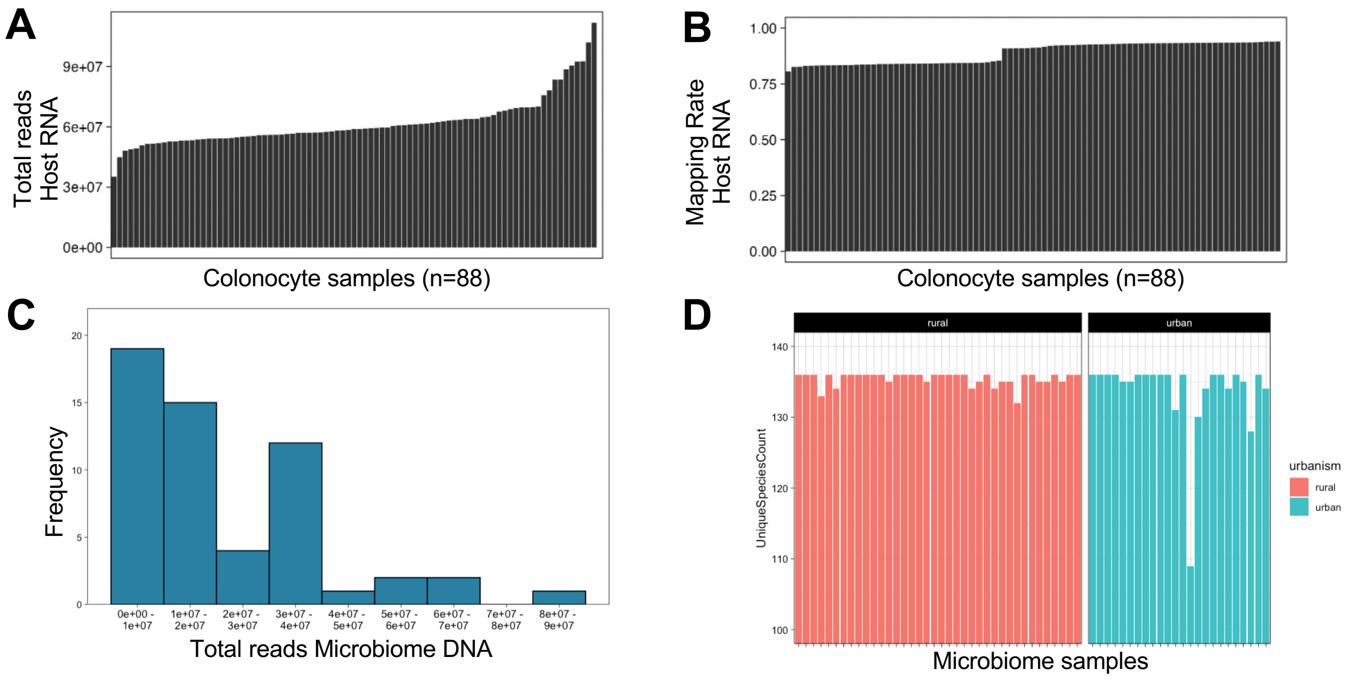

FIGURE S1 Relevant for Figure 1 and Methods. QC and differential abundance of microbiome data. **A** Total reads for host colonocyte RNA-sequencing **B** Mapping rate of host colonocyte RNA-sequencing **C** Total reads for metagenomic shotgun sequencing, ranging 3.5 to 87 million reads. **D** Total number of unique microbial species assigned to each sample.

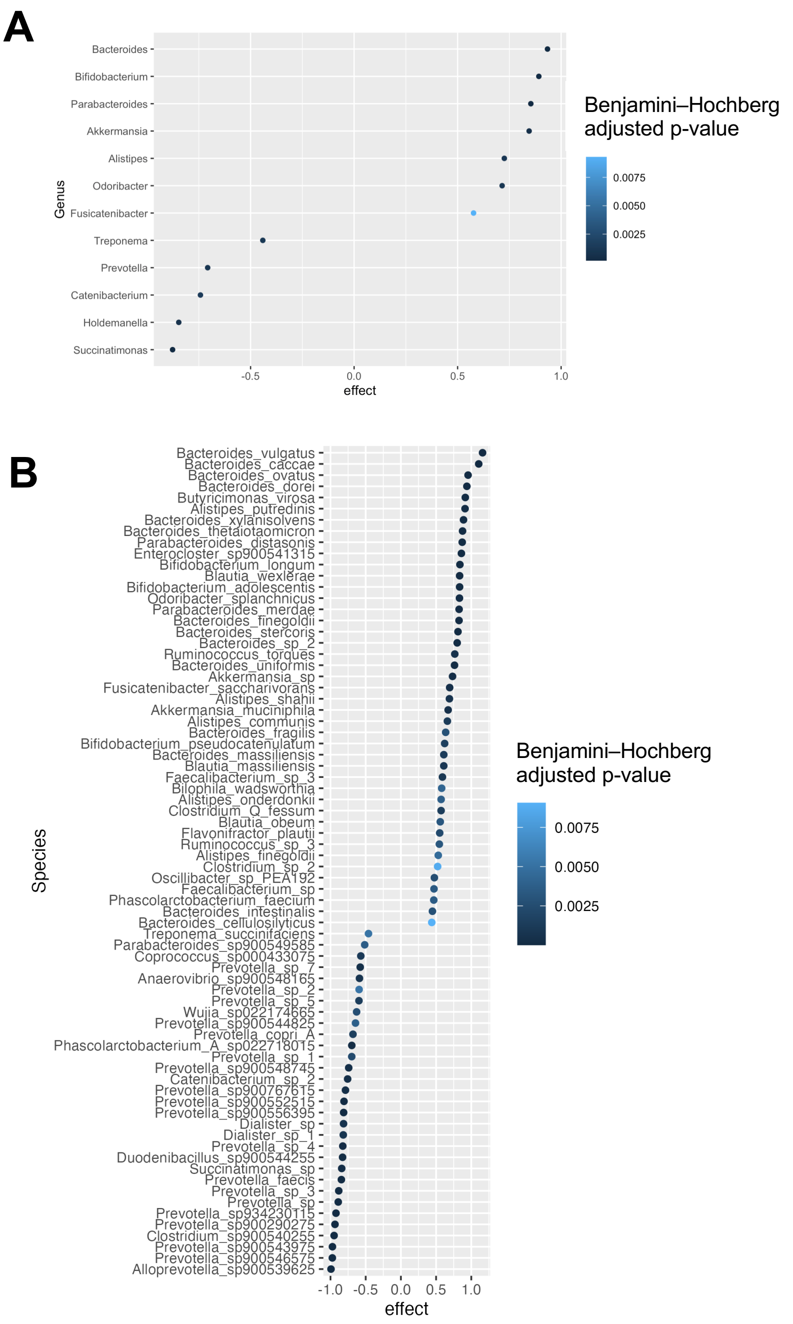

FIGURE S2 Relevant for Figure 1. Taxonomic differential abundance. **A**, **B** Differential abundance analysis of microbiome samples in urban v. rural populations, shown at the genus (A) and species (B) level. Effects > 0 reflect taxa that are enriched in urban populations, < 0 are those enriched in rural populations. Only significant (FDR < 0.1) results are shown.

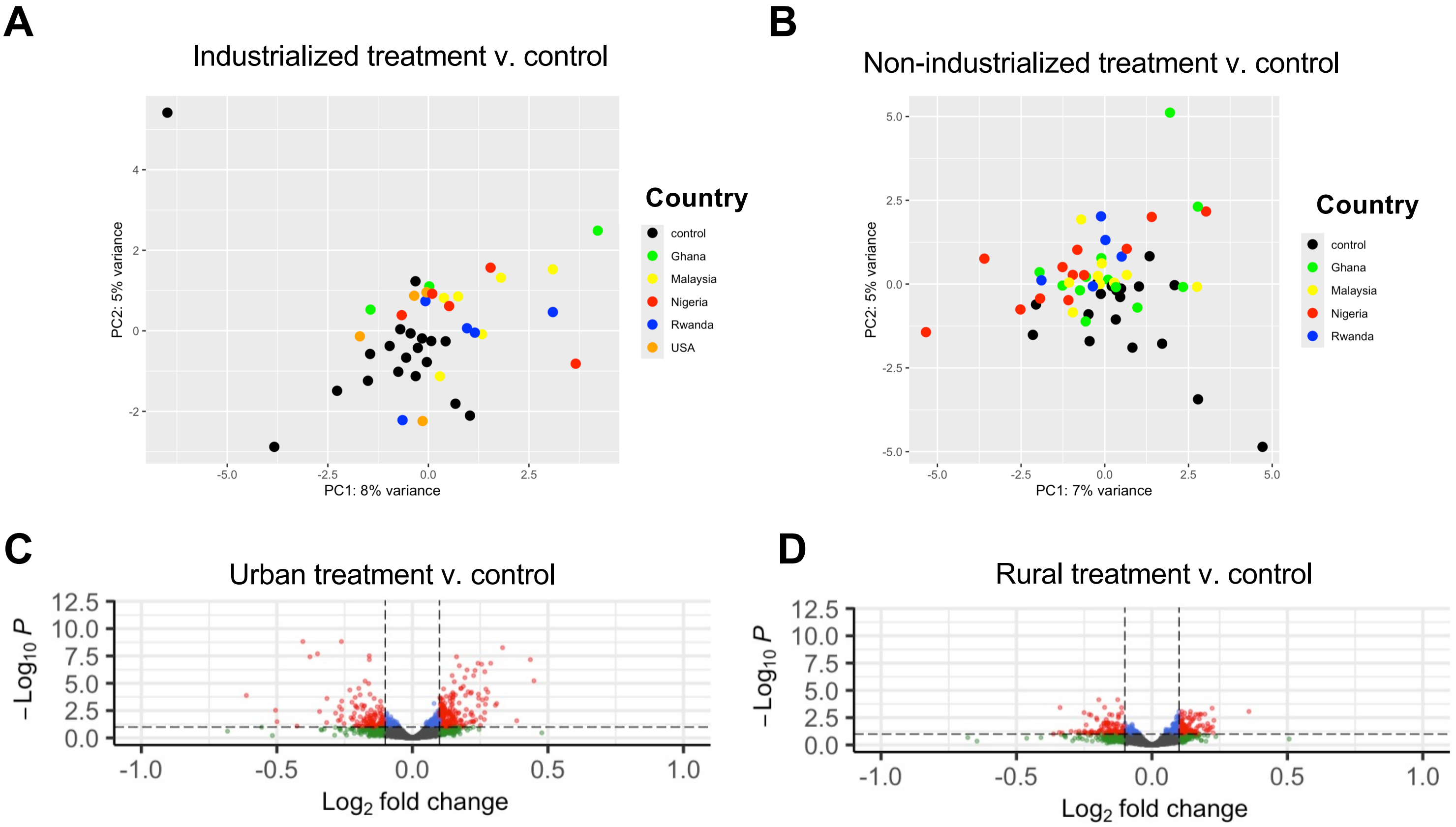

FIGURE S3 Relevant for Figure 2. **A, B** PCA plot depicting gene expression of colonocyte samples. Black dots indicate untreated controls. A, colored dots represent colonocytes treated with urban microbiomes from Ghana, Malaysia, Nigeria, Rwanda, and the USA (green, yellow, red, blue, orange, respectively). B, same as A, but colored dots are colonocytes treated with rural microbiomes. **C, D** Volcano plots showing host gene expression in response to urban microbiome versus control (C) and rural microbiome treatment versus control (D). Log2 fold change value (x axis) and *p*-value (y axis) results from differential expression analysis between treatments.

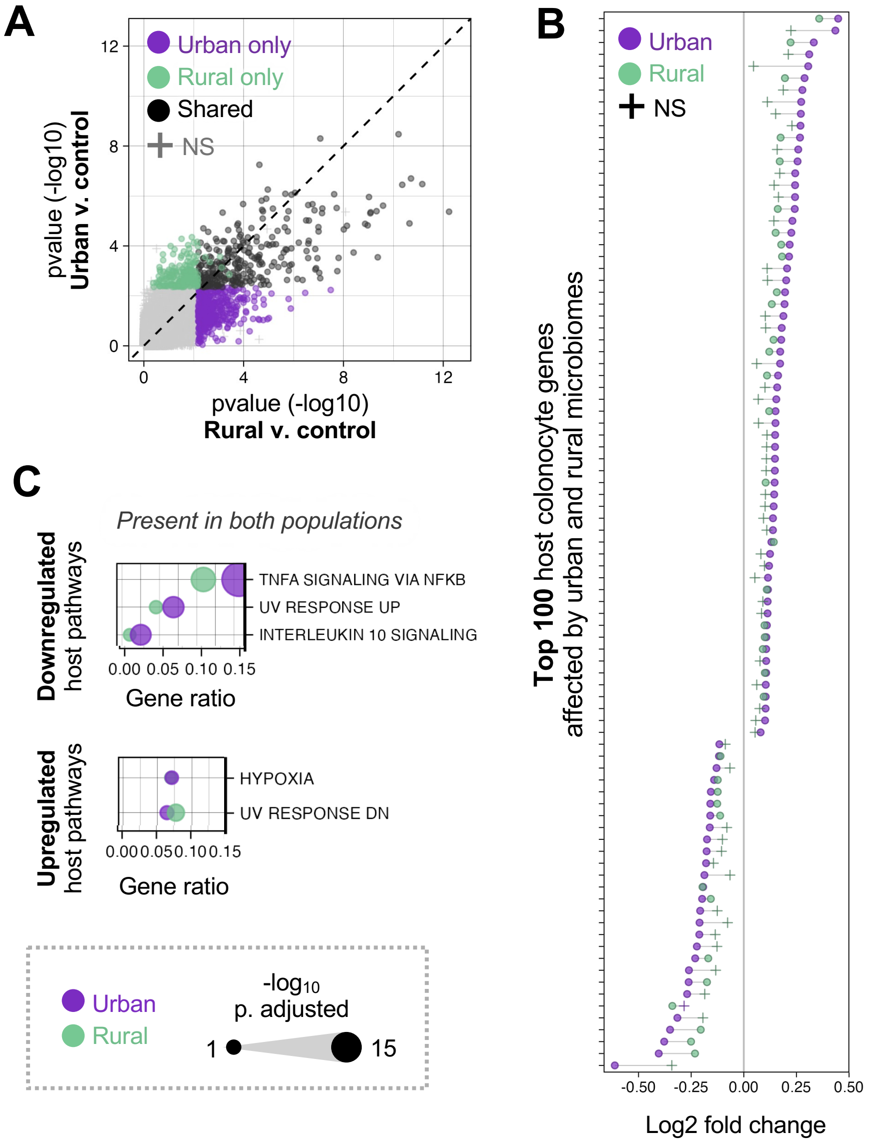

FIGURE S4 Relevant for Figure 2. **A DEGs compared to control condition.** Left: Raw p-values of host DEGs in response to urban (x-axis) and rural (y-axis) microbiomes. Dots indicate significant DEGs (FDR < 1x10-1) in urban only (purple), rural only (green), or both urban and rural (black). **B Top 100 host DEGs**. Subset of 100 host genes with lowest FDR-corrected *p* values and greatest absolute log2 fold changes in response to either urban (purple) or rural (green) microbiomes. Circles indicate significant (FDR < $1x10^{-1}$) genes, crosses represent not significant (NS) genes. **C Significant (FDR < $1x10^{-1}$, gene count ≥ 3) host gene pathways enriched in response to microbiome conditions.** Pathways that are found enriched in response to both urban and rural treatments shown. Down- and upregulated pathways are shown in the top and bottom panels, respectively. Dot size indicates -log10 (*p*-value). Urban and rural treatments produced equal values for the gene ratio for the host pathway of hypoxia.

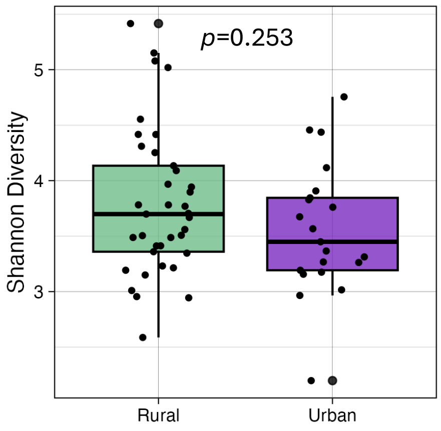

FIGURE S5 Relevant for Figure 2. Shannon diversity values among rural and urban microbiomes, respectively. Shannon diversity did not differ significantly between rural and urban microbiomes (Wilcoxon rank-sum test, p = 0.253).

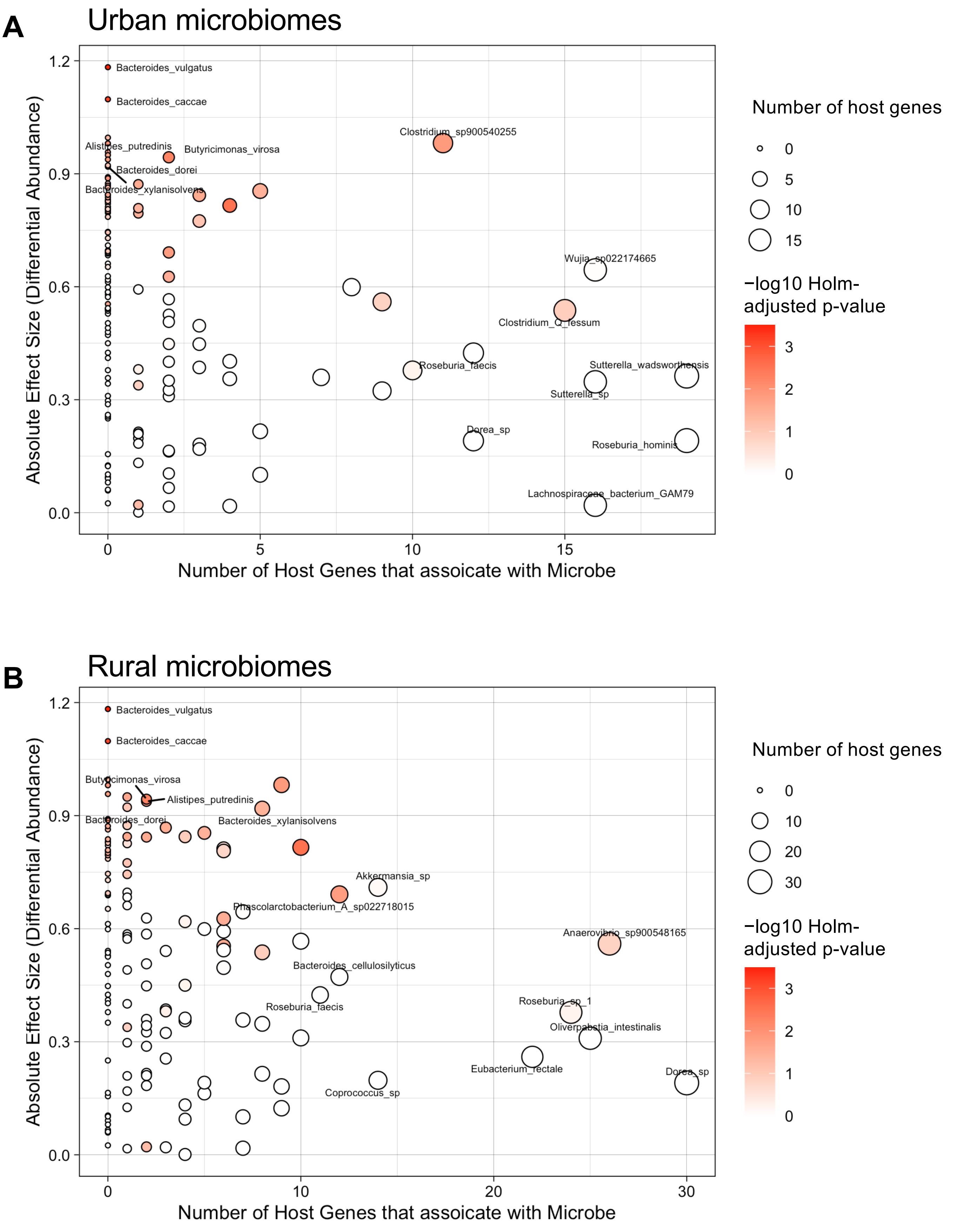
A
Urban microbiomes
Absolute Effect Size (Differential Abundance)
Number of Host Genes that assoicate with Microbe
Bacteroides_vulgatus
Bacteroides_caccae
Alistipes_putredinis
Butyricimonas_virosa
Bacteroides_dorei
Bacteroides_xylanisolvens
Clostridium_sp900540255
Wujia_sp022174665
Clostridium_Q_fessum
Roseburia_faecis
Sutterella_wadsworthensis
Sutterella_sp
Dorea_sp
Roseburia_hominis
Lachnospiraceae_bacterium_GAM79
Number of host genes
−log10 Holm-adjusted p-value
B
Rural microbiomes
Absolute Effect Size (Differential Abundance)
Number of Host Genes that assoicate with Microbe
Bacteroides_vulgatus
Bacteroides_caccae
Butyricimonas_virosa
Alistipes_putredinis
Bacteroides_dorei
Bacteroides_xylanisolvens
Akkermansia_sp
Phascolarctobacterium_A_sp022718015
Anaerovibrio_sp900548165
Bacteroides_cellulosilyticus
Roseburia_faecis
Roseburia_sp_1
Oliverpabstia_intestinalis
Eubacterium_rectale
Dorea_sp
Coprococcus_sp
Number of host genes
−log10 Holm-adjusted p-value

FIGURE S6 Relevant for Figure 3. Relationship between differential abundance of microbes in urban and rural microbiomes and their effect on host gene expression. **A** Circles represent all microbes found in urban microbiomes. x axis: absolute effect size from differential abundance calculation. y axis: number of host genes that each microbe associates with in pairwise lasso analysis. Shading of circle corresponds to holm adjusted *p*-value of differential abundance analysis. Microbes found in lower right quadrants have strong effects on host gene expression, but are not differentially abundant among urban and rural populations. Microbes found in top left quadrant are differentially abundant, but have minimal impact on host gene expression. **B** Same as A, but depict all microbes found in rural microbiomes.

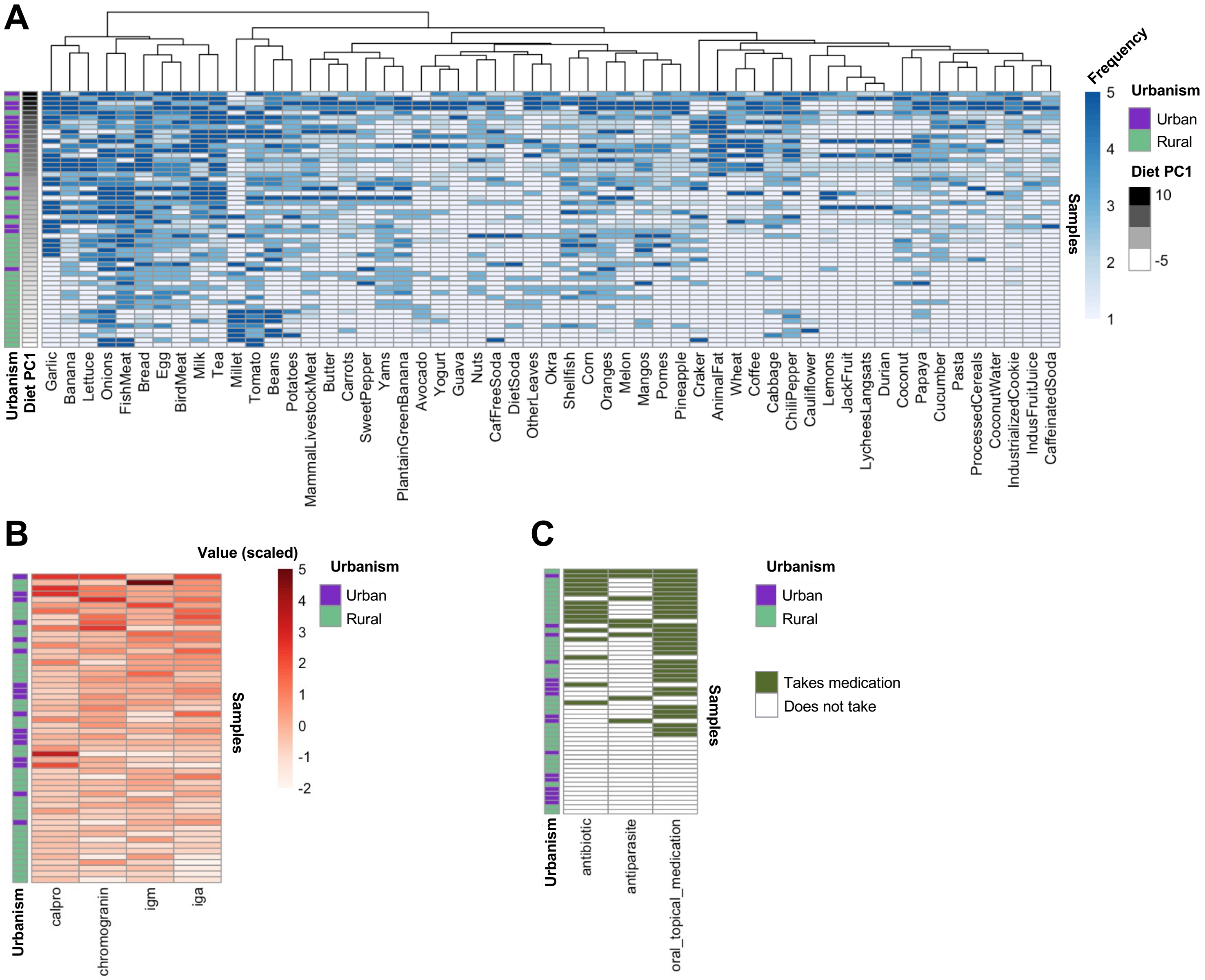

FIGURE S7 Relevant for Figure 4. **A** Heatmap of diet questionnaire responses (x axis) per participant (y axis). Frequency of consumption indicated by shading of blue tile. Hierarchical clustering applied to x axis. Further annotations provided in left panels: diet diversity (number of types of food consumed at least once), urbanism (green=rural, purple=urban), and value of PC1 diet. **B** Heatmap of blood markers (x axis) per participant (y axis). Scaled value of blood marker concentration indicated by shading of red tile. Further annotations provided in left panels: zscore of summed scaled blood marker value, urbanism. **C** Heatmap of medication use (x axis) per participant (y axis). Participants who take medication indicated by green tile; those who do not take medication indicated by white tile. Further annotations provided in left panels: medication diversity (number of classes of medications used by participant) and urbanism.

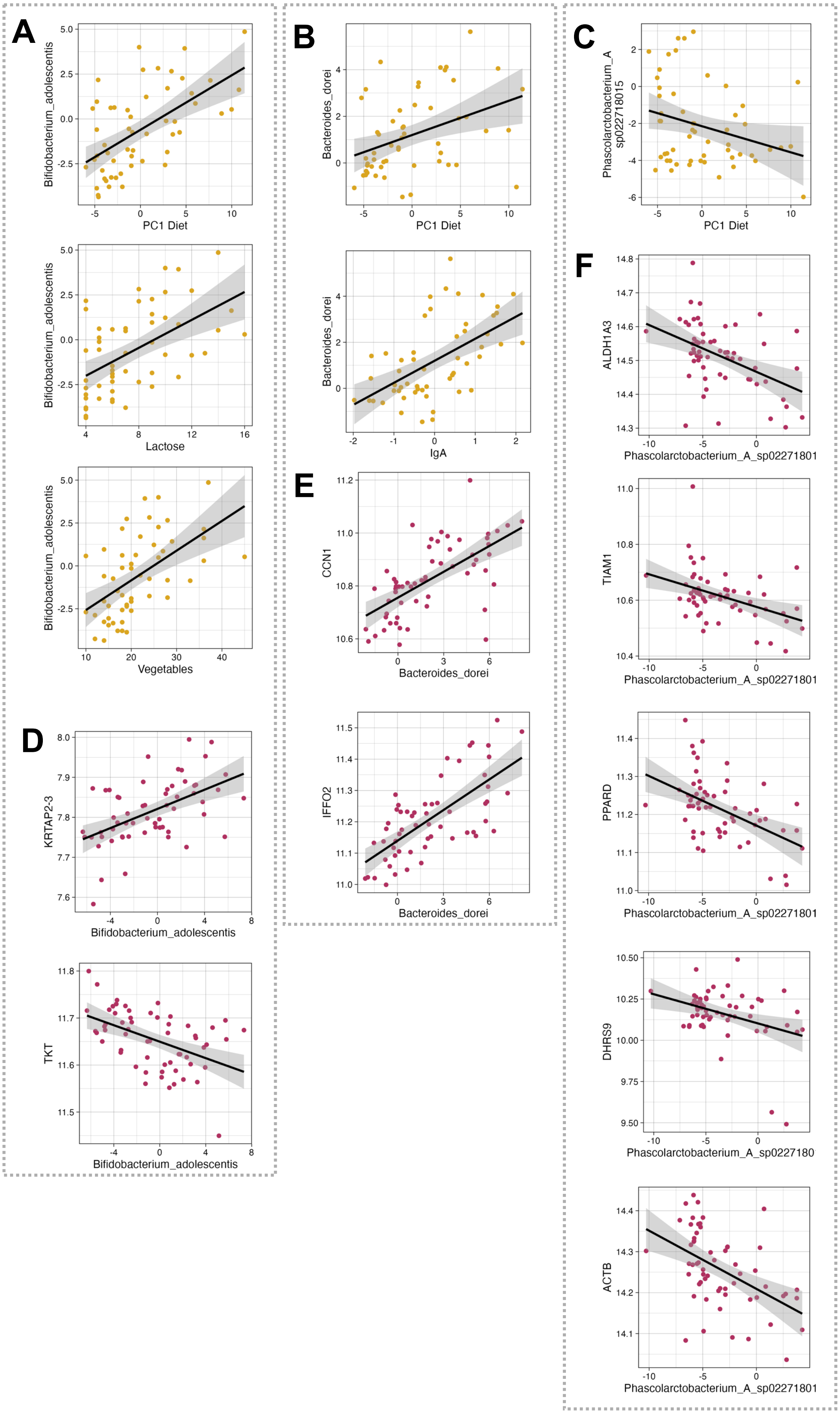

FIGURE S8 Relevant for Figure 4. **A-C** Scatterplot of lifestyle frequency (x-axis; **Methods**) and microbe abundance (CLR-transformed; y-axis) for *Bifidobacterium adolescentis, Bacteroides dorei,* and *Phascolarctobacterium A sp022718015,* respectively. **D-F** Scatterplot of microbe abundance (CLR-transformed; x-axis) and host gene expression (DESeq2 VST-transformed expression, **Methods**; y-axis) for *Bifidobacterium adolescentis, Bacteroides dorei,* and *Phascolarctobacterium A sp022718015,* respectively.

SUPPLEMENTARY TABLES

TABLE S1 Relevant for Figure 1. Participant metadata.

database	country	ethnicity	n urban	n rural	n male n female	age range
gmbc	Malaysia	jahai;thai;batek;chinese;indian	6	9	7_8	18_59
gmbc	Rwanda	rwandan	5	5	6_4	24_54
gmbc	Ghana	fante;ashanti;ga;ga_na;akan_fante;akan_akuapem	3	12	5_10	23_55
gmbc	Nigeria	yoruba;igbo;ibibio;efik	5	12	9_8	19_59
openbiome	USA	unknown	5	0	unknown	unknown

TABLE S2 Relevant for Figure 3. Number of associations of microbes with host genes.

taxa	Urban	Rural	All
Roseburia_sp_1	10	24	47
Veillonella	15	10	36
Anaerovibrio_sp900548165	9	26	36
Phascolarctobacterium_A_sp022718015	2	12	31
Dorea_sp	12	30	28
Roseburia_faecis	12	11	20
Roseburia_hominis	19	5	19
Anaerobutyricum	13	20	18
Clostridium_sp_3	2	5	16
Ruminococcus_bromii	2	2	15
Wujia_sp022174665	16	7	14
Oliverpabstia_intestinalis	2	25	13
Dorea_formicigenerans	4	7	13
Ruminococcus_sp_3		3	12
Clostridium_Q_fessum	15	8	12
Sutterella_sp	16	8	11
Enterobacter	1	1	11
Veillonellales	2	3	10
Duodenibacillus_sp900544255	3	2	10
Sutterella_wadsworthensis	19	4	8
Ruminococcus_E_sp003526955	1	8	8
Coprococcus_sp	1	14	8
Ruminococcus_bicirculans	1	4	7
Phascolarctobacterium_faecium	3	6	7
Eggerthellaceae	2	6	7
Coprococcus_comes	5	7	6
Butyricicoccus	2	4	6
Akkermansia_sp		14	6
Phocaeicola_sp000434735	3	3	5
Phascolarctobacterium_succinatutens	7	2	5
Lachnospiraceae_bacterium_GAM79	16	3	5
Erysipelatoclostridium		3	5
Dorea_longicatena		3	5
Catenibacterium_sp	3	9	5
Butyricimonas	4	2	5
Betaproteobacteria		2	5
Sutterella	11		4
Ruminococcus_faecis	1	4	4

Eubacterium_rectale		22	4
Eisenbergiella_sp900066775	1	1	4
Coprococcus_sp000433075		6	4
Clostridium_sp_2		6	4
Blautia_massiliensis		4	4
Bifidobacterium_pseudocatenulatum	8	5	4
Roseburia_inulinivorans	5	2	3
Roseburia_intestinalis	4	4	3
Prevotella_sp900544825		1	3
Oscillibacter_sp			3
Hominisplanchenecus_faecis	9	3	3
Gemmiger	1		3
Clostridium_innocuum		10	3
Ruminococcus_sp_4	2	1	2
Ruminococcus_sp_2	3	1	2
Ruminococcus			2
Roseburia		1	2
Phascolarctobacterium	2		2
Parabacteroides_sp900549585	2		2
Negativicutes		2	2
Megasphaera		4	2
Firmicutes			2
Faecalibacterium_sp_4		9	2
Faecalibacterium_sp_3		2	2
Faecalibacterium_sp		1	2
Eubacterium_sp_5			2
Dorea	1		2
Dialister	1		2
Cryptobacteroides_sp000431015	2	2	2
Coprobacillus	6	1	2
Clostridium_sp900540255	11	9	2
Clostridium	1	14	2
Catenibacterium_sp_2		1	2
Burkholderiales			2
Bifidobacterium_adolescentis	5	5	2
Barnesiella	1	1	2
Bacteroides_plebeius	1	1	2
Bacteroides_fragilis		2	2
Bacteroides_finegoldii	4	10	2
Bacteroides_dorei		1	2
Alloprevotella_sp900539625			2
Alistipes_senegalensis	2		2
Alistipes_onderdonkii	1	6	2

Alistipes_finegoldii			2
Vescimonas_sp900551995	2		1
Veillonellaceae	1		1
Sutterellaceae			1
Streptomycetales			1
Streptomyces	2		1
Streptococcaceae		1	1
Ruminococcus_torques	3	1	1
Proteobacteria		5	1
Prevotella_sp_3			1
Prevotella_faecis		3	1
Oscillibacter_sp_1	2		1
Holdemanella_sp_1			1
Holdemanella		1	1
Gemmiger_qucibialis	4		1
Gammaproteobacteria		2	1
Flavobacteriales	1		1
Flavobacteriaceae	2		1
Faecalibacterium_prausnitzii		2	1
Eubacterium_sp_1	1	2	1
Eubacterium			1
Erysipelotrichales		4	1
Dysosmobacter_sp900544615		4	1
Dialister_sp_1		6	1
Dialister_sp		1	1
Deltaproteobacteria			1
Coriobacteriia	1		1
Coriobacteriales			1
Collinsella_aerofaciens			1
Clostridia			1
Blautia_obeum	2	10	1
Barnesiellaceae			1
Barnesiella_intestinihominis	3		1
Bacteroides_sp_2		6	1
Bacteroides_massiliensis	2	6	1
Bacteroides_intestinalis	2	2	1
Bacteroides_cellulosilyticus		12	1
Alistipes_putredinis		2	1
Aeromonadales	1		1
Acetatifactor_sp900066565	2		1
Spirochaetales		1	
Selenomonadales		4	
Selenomonadaceae		3	
Prevotella_sp900290275		1	

Prevotella_sp_7		1	
Prevotella_sp_5		1	
Prevotella_sp_1		1	
Prevotella_sp		1	
Prevotella_copri_A		1	
Prevotella_copri		1	
Porphyromonadaceae	1		
Parabacteroides_merdae	1		
Parabacteroides_distasonis		1	
Lactobacillales		1	
Lactobacillaceae		2	
Lachnospira_eligens		1	
Klebsiella_pneumoniae	1	2	
Klebsiella		1	
Gemmiger_formicilis	2	1	
Fusicatenibacter	1		
Flavonifractor		1	
Flavobacteriia	2		
Faecalibacterium_sp_5		2	
Eubacterium_sp_4	1	2	
Escherichia		1	
Erysipelotrichia		5	
Erysipelotrichaceae	1	9	
Enterococcus		1	
Enterococcaceae		1	
Enterocloster_sp900541315	1		
Enterobacterales		2	
Copromonas_sp900066785		7	
Coprococcus_sp_4	1	3	
Coprococcus_sp_3		4	
Coprococcus_eutactus		1	
Coprococcus		2	
Collinsella		1	
Clostridiaceae		1	
Butyricimonas_virosa	2	2	
Bifidobacterium_longum		4	
Bifidobacteriales	3		
Bacteroides_xylanisolvens		8	
Bacteroides_stercoris	1		
Bacilli		1	
Bacillaceae		1	
Anaerostipes		1	
Acidaminococcus		11	

TABLE S3 Relevant for Figure 3. Number of associations of microbes with host pathways.

microbial_function	Urban	Rural	All
mc_PWY_5676__acetyl_CoA_fermentation_to_butanoate_II	41	6	35
mc_PWY_1861__formaldehyde_assimilation_II__assimilatory_RuMP_Cycle_	31	44	86
mc_PWY_5189__tetrapyrrole_biosynthesis_II__from_glycine_	22	1	51
mc_PWY_7883__anhydromuropeptides_recycling_II	18	5	40
mc_GLUDEG_I_PWY__GABA_shunt	14	4	34
mc_PWY_5005__biotin_biosynthesis_II	13	27	132
mc_PWY_622__starch_biosynthesis	10	6	56
mc_PWY_7013___S__propane_1_2_diol_degradation	10	9	179
mc_LACTOSECAT_PWY__lactose_and_galactose_degradation_I	8	19	51
mc_PWY_4041___gamma__glutamyl_cycle	8	3	19
mc_PWY_7185__UTP_and_CTP_dephosphorylation_I	8	2	8
mc_ARG_POLYAMINE_SYN__superpathway_of_arginine_and_polyamine_biosynthesis	7		3
mc_PWY_7254__TCA_cycle_VII__acetate_producers_	7	60	202
mc_P441_PWY__superpathway_of_N_acetylneuraminate_degradation	6		8
mc_PWY_5971__palmitate_biosynthesis__type_II_fatty_acid_synthase_	6		13
mc_PWY_5981__CDP_diacylglycerol_biosynthesis_III	6	11	68
mc_PWY_7942__5_oxo_L_proline_metabolism	6	3	15
mc_PWY_5505__L_glutamate_and_L_glutamine_biosynthesis	5	3	20
mc_PWY_5690__TCA_cycle_II__plants_and_fungi_	5	27	115
mc_PWY1ZNC_1__assimilatory_sulfate_reduction_IV	4	2	38
mc_PWY_5913__partial_TCA_cycle__obligate_autotrophs_	4		12
mc_PWY_6590__superpathway_of_Clostridium_acetobutylicum_acidogenic_fermentation	4	4	6
mc_PWY_6703__preQ0_biosynthesis	4		
mc_PWY_702__L_methionine_biosynthesis_II	4		7
mc_DENOVOPURINE2_PWY__superpathway_of_purine_nucleotides_de_novo_biosynthesis_II	3		3
mc_PWY_6630__superpathway_of_L_tyrosine_biosynthesis	3		4
mc_PWY_7234__inosine_5__phosphate_biosynthesis_III	3	2	11
mc_PWY_8131__5__deoxyadenosine_degradation_II	3	20	66
mc_GLUCOSE1PMETAB_PWY__glucose_and_glucose_1_phosphate_degradation	2	3	25
mc_PPGPPMET_PWY__ppGpp_metabolism	2	3	54
mc_PWY0_162__superpathway_of_pyrimidine_ribonucleotides_de_novo_biosynthesis	2	1	3
mc_PWY_5136__fatty_acid__beta__oxidation_II__plant_peroxisome_	2		2
mc_PWY_5367__petroselinate_biosynthesis	2	6	5
mc_PWY_6305__superpathway_of_putrescine_biosynthesis	2	9	11

mc_PWY_6607__guanosine_nucleotides_degradation_I	2	3	10
mc_PWY_7118__chitin_deacetylation	2		12
mc_CENTFERM_PWY__pyruvate_fermentation_to_butanoate	1	3	10
mc_GLYCOCAT_PWY__glycogen_degradation_I	1	4	23
mc_GOLPDLCAT_PWY__superpathway_of_glycerol_degradation_to_1_3_propanediol	1		9
mc_HEMESYN2_PWY__heme_b_biosynthesis_II__oxygen_independent_	1	6	28
mc_P108_PWY__pyruvate_fermentation_to_propanoate_I	1	4	10
mc_P124_PWY__Bifidobacterium_shunt	1	17	10
mc_P161_PWY__acetylene_degradation__anaerobic_	1		
mc_POLYAMINSYN3_PWY__superpathway_of_polyamine_biosynthesis_II	1	26	68
mc_PRPP_PWY__superpathway_of_histidine__purine__and_pyrimidine_biosynthesis	1	2	10
mc_PWY0_1261__anhydromuropeptides_recycling_I	1	3	10
mc_PWY0_1298__superpathway_of_pyrimidine_deoxyribonucleosides_degradation	1	1	5
mc_PWY66_409__superpathway_of_purine_nucleotide_salvage	1		10
mc_PWY_6470__peptidoglycan_biosynthesis_V___beta__lactam_resistance_	1	18	34
mc_PWY_6545__pyrimidine_deoxyribonucleotides_de_novo_biosynthesis_III	1		
mc_PWY_6895__superpathway_of_thiamine_diphosphate_biosynthesis_II	1	14	79
mc_PWY_6936__seleno_amino_acid_biosynthesis__plants_	1		
mc_PWY_7115__C4_photosynthetic_carbon_assimilation_cycle__NAD_ME_type	1	2	4
mc_PWY_7210__pyrimidine_deoxyribonucleotides_biosynthesis_from_CTP	1	5	3
mc_PWY_7315__dTDP_N_acetylthomosamine_biosynthesis	1	21	83
mc_PWY_7356__thiamine_diphosphate_salvage_IV__yeast_	1	5	9
mc_PWY_7383__anaerobic_energy_metabolism__invertebrates__cytosol_	1	4	21
mc_PWY_7761__NAD_salvage_pathway_II__PNC_IV_cycle_	1		
mc_PWY_8187__L_arginine_degradation_XIII__reductive_Stickland_reaction_	1	3	3
mc_PWY_I9__L_cysteine_biosynthesis_VI__from_L_methionine_	1	2	28
mc_PWY_5130__2_oxobutanoate_degradation_I		68	128
mc_PWY_6612__superpathway_of_tetrahydrofolate_biosynthesis		41	44
mc_PWY_6892__thiazole_component_of_thiamine_diphosphate_biosynthesis_I		38	4
mc_PWY_6906__chitin_derivatives_degradation		26	20
mc_PWY_7198__pyrimidine_deoxyribonucleotides_de_novo_biosynthesis_IV		26	157
mc_FUCCAT_PWY__fucose_degradation		25	51
mc_PWY_5030__L_histidine_degradation_III		19	18
mc_PWY0_1061__superpathway_of_L_alanine_biosynthesis		18	43
mc_PWY_6168__flavin_biosynthesis_III__fungi_		17	12
mc_FERMENTATION_PWY__mixed_acid_fermentation		14	14

mc_PWY0_1477__ethanolamine_utilization		14	21
mc_P42_PWY__incomplete_reductive_TCA_cycle		12	53
mc_HEME_BIOSYNTHESIS_II__heme_b_biosynthesis_I__aerobic_		9	11
mc_PWY_7211__superpathway_of_pyrimidine_deoxyribonucleotides_de_novo_biosynthesis		9	31
mc_DAPLYSINESYN_PWY__L_lysine_biosynthesis_I		7	19
mc_ORNDEG_PWY__superpathway_of_ornithine_degradation		7	8
mc_PWY_6859__all_trans_farnesol_biosynthesis		7	12
mc_PWY_6902__chitin_degradation_II__Vibrio_		7	15
mc_PWY_621__sucrose_degradation_III__sucrose_invertase_		6	9
mc_GLUCARGALACTSUPER_PWY__superpathway_of_D_glucarate_and_D_galactarate_degradation		5	9
mc_METH_ACETATE_PWY__methanogenesis_from_acetate		5	5
mc_P105_PWY__TCA_cycle_IV__2_oxoglutarate_decarboxylase_		5	86
mc_PWY_6595__superpathway_of_guanosine_nucleotides_degradation__plants_		5	6
mc_GLCMANNANAUT_PWY__superpathway_of_N_acetylglucosamine__N_acetylmannosamine_and_N_acetylneuraminate_degradation		4	5
mc_HISDEG_PWY__L_histidine_degradation_I		4	6
mc_PWY_5497__purine_nucleobases_degradation_II__anaerobic_		4	9
mc_PWY_6147__6_hydroxymethyl_dihydropterin_diphosphate_biosynthesis_I		4	9
mc_PWY_6731__starch_degradation_III		4	
mc_PWY_7392__taxadiene_biosynthesis__engineered_		4	10
mc_GALACT_GLUCUROCAT_PWY__superpathway_of_hexuronide_and_hexuronate_degradation		3	15
mc_P164_PWY__purine_nucleobases_degradation_I__anaerobic_		3	8
mc_PWY0_862___5Z__dodecenoate_biosynthesis_I		3	26
mc_PWY_5121__superpathway_of_geranylgeranyl_diphosphate_biosynthesis_II__via_MEP_		3	
mc_PWY_6606__guanosine_nucleotides_degradation_II		3	13
mc_PWY_6608__guanosine_nucleotides_degradation_III		3	8
mc_SALVADEHYPOX_PWY__adenosine_nucleotides_degradation_II		3	7
mc_TCA__TCA_cycle_I__prokaryotic_		3	
mc_ARGININE_SYN4_PWY__L_ornithine_biosynthesis_II		2	3
mc_CITRULBIO_PWY__L_citrulline_biosynthesis		2	3
mc_GALACTARDEG_PWY__D_galactarate_degradation_I		2	1
mc_PWY0_1241__ADP_L_glycero__beta__D_manno_heptose_biosynthesis		2	15
mc_PWY0_1297__superpathway_of_purine_deoxyribonucleosides_degradation		2	9
mc_PWY_5659__GDP_mannose_biosynthesis		2	
mc_PWY_5862__superpathway_of_demethylmenaquinol_9_biosynthesis		2	2
mc_PWY_6803__phosphatidylcholine_acyl_editing		2	5
mc_PWY_7345__superpathway_of_anaerobic_sucrose_degradation		2	
mc_PWY_821__superpathway_of_sulfur_amino_acid_biosynthesis__Saccharomyces_cerevisiae_		2	
mc_FOLSYN_PWY__superpathway_of_tetrahydrofolate_biosynthesis_and_salvage		1	6
mc_FUC_RHAMCAT_PWY__superpathway_of_fucose_and_rhamnose_degradation		1	4

mc_GALACTUROCAT_PWY__D_galacturonate_degradation_I		1	5
mc_POLYAMSYN_PWY__superpathway_of_polyamine_biosynthesis_I		1	27
mc_PWY0_1479__tRNA_processing		1	4
mc_PWY_4984__urea_cycle		1	2
mc_PWY_5675__nitrate_reduction_V__assimilatory_		1	
mc_PWY_5850__superpathway_of_menaquinol_6_biosynthesis		1	5
mc_PWY_6285__superpathway_of_fatty_acids_biosynthesis__E__coli_		1	3
mc_PWY_6531__mannitol_cycle		1	5
mc_PWY_6969__TCA_cycle_V__2_oxoglutarate_synthase_		1	5
mc_PWY_7184__pyrimidine_deoxyribonucleotides_de_novo_biosynthesis_I		1	
mc_PWY_7328__superpathway_of_UDP_glucose_derived_O_antigen_building_blocks_biosynthesis		1	2
mc_PWY_8073__lipid_IVA_biosynthesis__P__putida_		1	1
mc_PWY_2941__L_lysine_biosynthesis_II			19
mc_PWY_6549__L_glutamine_biosynthesis_III			11
mc_PWY_5896__superpathway_of_menaquinol_10_biosynthesis			5
mc_GLYOXYLATE_BYPASS__glyoxylate_cycle			4
mc_PWY_5918__superpathway_of_heme_b_biosynthesis_from_glutamate			4
mc_SULFATE_CYS_PWY__superpathway_of_sulfate_assimilation_and_cysteine_biosynthesis			4
mc_PWY3O_4107__NAD_salvage_pathway_V__PNC_V_cycle_			3
mc_PYRIDNUCSAL_PWY__NAD_salvage_pathway_I__PNC_VI_cycle_			3
mc_P461_PWY__hexitol_fermentation_to_lactate__formate__ethanol_and_acetate			2
mc_PWY66_399__gluconeogenesis_III			2
mc_PWY_5837__2_carboxy_1_4_naphthoquinol_biosynthesis			2
mc_PWY_5840__superpathway_of_menaquinol_7_biosynthesis			2
mc_PWY_6628__superpathway_of_L_phenylalanine_biosynthesis			2
mc_ASPASN_PWY__superpathway_of_L_aspartate_and_L_asparagine_biosynthesis			1
mc_COBALSYN_PWY__superpathway_of_adenosylcobalamin_salvage_from_cobinamide_I			1
mc_GLYCOLYSIS_E_D__superpathway_of_glycolysis_and_the_Entner_Doudoroff_pathway			1
mc_PWY0_166__superpathway_of_pyrimidine_deoxyribonucleotides_de_novo_biosynthesis__E__coli_			1
mc_PWY0_301__L_ascorbate_degradation_I__bacterial__anaerobic_			1
mc_PWY_5188__tetrapyrrole_biosynthesis_I__from_glutamate_			1
mc_PWY_5384__sucrose_degradation_IV__sucrose_phosphorylase_			1
mc_PWY_5989__stearate_biosynthesis_II__bacteria_and_plants_			1
mc_PWY_6270__isoprene_biosynthesis_I			1
mc_PWY_6284__superpathway_of_unsaturated_fatty_acids_biosynthesis__E__coli_			1
mc_PWY_6353__purine_nucleotides_degradation_II__aerobic_			1
mc_PWY_6823__molybdopterin_biosynthesis			1
mc_PWY_7117__C4_photosynthetic_carbon_assimilation_cycle__PEPCK_type			1
mc_PWY_7197__pyrimidine_deoxyribonucleotide_phosphorylation			1

mc_PWY_7400__L_arginine_biosynthesis_IV__archaebacteria_			1
mc_PWY_7858___5Z__dodecenoate_biosynthesis_II			1
mc_THISYNARA_PWY__superpathway_of_thiamine_diphosphate_biosynthesis_III__eukaryotes_			1

TABLE S4 Relevant for Figure 3. Clusters of correlated host genes/pathways and microbes.

Host gene & Microbes

Urban		
Cluster	*microbe_taxa_cluster*	*n_host_genes*
2	Spirochaetaceae; Treponema	377
3	Coprobacillus; Roseburia_sp_1	386
4	Flavobacteriia; Flavobacteriaceae	370
5	Klebsiella; Klebsiella_pneumoniae	390
Rural		
Cluster	*microbe_taxa_cluster*	*n_host_genes*
1	Akkermansiaceae; Akkermansia	389
2	Bacteroides_finegoldii; Parabacteroides_merdae	399
3	Clostridiaceae; Clostridium	397
4	Erysipelotrichia; Erysipelotrichales	384
8	Enterobacter; Klebsiella; Anaerobutyricum; Klebsiella_pneumoniae	386
9	Dialister; Dialister_sp	394
10	Coprococcus; Coprococcus_sp	405

Host gene & Microbe pathways

Urban		
cluster	*microbe_function_cluster*	*n_host_genes*
3	P124_PWY__Bifidobacterium_shunt; mc_PWY_5676__acetyl_CoA_fermentation_to_butanoate_II	221
4	PWY_7118__chitin_deacetylation; PWY_8131__5__deoxyadenosine_degradation_II	231
10	PPGPPMET_PWY__ppGpp_metabolism	239
Rural		
cluster	*microbe_function_cluster*	*n_host_genes*
1	HSERMETANA_PWY__L_methionine_biosynthesis_III; PWY_7221__guanosine_ribonucleotides_de_novo_biosynthesis; PWY_7977__L_methionine_biosynthesis_IV	233
4	PPGPPMET_PWY__ppGpp_metabolism; PWY_6731__starch_degradation_III	231
6	ARGININE_SYN4_PWY__L_ornithine_biosynthesis_II; GLUDEG_I_PWY__GABA_shunt	247
9	P164_PWY__purine_nucleobases_degradation_I__anaerobic_; PWY0_862___5Z__dodecenoate_biosynthesis_I	231
10	PWY_6607__guanosine_nucleotides_degradation_I	236

TABLE S5 Relevant for Figure 2. Host pathways upregulated in response to low diversity and high diversity microbiomes.

Host pathway	GeneRatio	BgRatio	pvalue	p.adjust	Enriched in
REACTOME_GLUCONEOGENESIS	5/473	19/9087	2.37E-03	5.44E-02	Low Diversity
KEGG_MEDICUS_REFERENCE_MICROTUBULE_NUCLEATION	5/473	23/9087	5.77E-03	9.70E-02	Low Diversity
REACTOME_ROLE_OF_LAT2_NTAL_LAB_ON_CALCIUM_MOBILIZATION	4/364	14/9087	1.84E-03	5.42E-02	High Diversity
REACTOME_A_TETRASACCHARIDE_LINKER_SEQUENCE_IS_REQUIRED_FOR_GAG_SYNTHESIS	6/473	22/9087	7.04E-04	2.83E-02	Low Diversity
KEGG_MEDICUS_REFERENCE_ITGA_B_FAK_CDC42_SIGNALING_PATHWAY	6/473	24/9087	1.16E-03	3.84E-02	Low Diversity
KEGG_MEDICUS_REFERENCE_ITGA_B_RHOG_RAC_SIGNALING_PATHWAY	6/473	26/9087	1.82E-03	4.83E-02	Low Diversity
REACTOME_MET_ACTIVATES_PTK2_SIGNALING	6/473	26/9087	1.82E-03	4.83E-02	Low Diversity
REACTOME_SYNDECAN_INTERACTIONS	6/473	26/9087	1.82E-03	4.83E-02	Low Diversity
PID_SYNDECAN_4_PATHWAY	6/473	27/9087	2.24E-03	5.36E-02	Low Diversity
KEGG_MEDICUS_REFERENCE_ARL8_REGULATED_MICROTUBULE_PLUS_END_DIRECTED_TRANSPORT	6/473	29/9087	3.29E-03	6.56E-02	Low Diversity
REACTOME_LAMININ_INTERACTIONS	6/473	29/9087	3.29E-03	6.56E-02	Low Diversity
REACTOME_METABOLISM_OF_FAT_SOLUBLE_VITAMINS	6/473	32/9087	5.49E-03	9.70E-02	Low Diversity
REACTOME_DOWNSTREAM_SIGNALING_OF_ACTIVATED_FGFR4	5/364	20/9087	9.45E-04	4.54E-02	High Diversity
REACTOME_DAP12_SIGNALING	5/364	21/9087	1.20E-03	4.54E-02	High Diversity
REACTOME_DOWNSTREAM_SIGNALING_OF_ACTIVATED_FGFR2	5/364	21/9087	1.20E-03	4.54E-02	High Diversity
BIOCARTA_VEGF_PATHWAY	5/364	22/9087	1.50E-03	5.02E-02	High Diversity
REACTOME_DOWNSTREAM_SIGNALING_OF_ACTIVATED_FGFR1	5/364	23/9087	1.86E-03	5.42E-02	High Diversity
PID_VEGFR1_PATHWAY	5/364	24/9087	2.27E-03	5.49E-02	High Diversity
REACTOME_SIGNALING_BY_ERBB2_IN_CANCER	5/364	25/9087	2.75E-03	6.12E-02	High Diversity
REACTOME_SIGNALING_BY_ALK	5/364	26/9087	3.29E-03	6.81E-02	High Diversity
REACTOME_DAP12_INTERACTIONS	5/364	27/9087	3.91E-03	7.47E-02	High Diversity
REACTOME_FLT3_SIGNALING_IN_DISEASE	5/364	27/9087	3.91E-03	7.47E-02	High Diversity
BIOCARTA_DEATH_PATHWAY	5/364	29/9087	5.39E-03	9.68E-02	High Diversity
REACTOME_SIGNALING_BY_CSF1_M_CSF_IN_MYELOID_CELLS	5/364	29/9087	5.39E-03	9.68E-02	High Diversity
PID_UPA_UPAR_PATHWAY	7/473	31/9087	8.74E-04	3.10E-02	Low Diversity
REACTOME_ELASTIC_FIBRE_FORMATION	7/473	35/9087	1.87E-03	4.83E-02	Low Diversity
PID_P38_ALPHA_BETA_DOWNSTREAM_PATHWAY	7/473	37/9087	2.61E-03	5.44E-02	Low Diversity
PID_THROMBIN_PAR1_PATHWAY	7/473	37/9087	2.61E-03	5.44E-02	Low Diversity
REACTOME_MET_PROMOTES_CELL_MOTILITY	7/473	37/9087	2.61E-03	5.44E-02	Low Diversity
REACTOME_DIFFERENTIATION_OF_KERATINOCYTES_IN_INTERFOLLICULAR_EPIDERMIS_IN_MAMMALIAN_SKIN	7/473	40/9087	4.14E-03	7.62E-02	Low Diversity
REACTOME_BMAL1_CLOCK_NPAS2_ACTIVATES_CIRCADIAN_GENE_EXPRESSION	6/364	25/9087	3.67E-04	3.55E-02	High Diversity
PID_TRAIL_PATHWAY	6/364	28/9087	7.06E-04	4.54E-02	High Diversity

PID_NEPHRIN_NEPH1_PATHWAY	6/364	29/9087	8.60E-04	4.54E-02	High Diversity
REACTOME_ANTIGEN_PRESENTATION_FOLDING_ASSEMBLY_AND_PEPTIDE_LOADING_OF_CLASS_I_MHC	6/364	29/9087	8.60E-04	4.54E-02	High Diversity
REACTOME_DOWNSTREAM_SIGNAL_TRANSDUCTION	6/364	29/9087	8.60E-04	4.54E-02	High Diversity
BIOCARTA_FCER1_PATHWAY	6/364	34/9087	2.06E-03	5.42E-02	High Diversity
REACTOME_SIGNALING_BY_FGFR4	6/364	34/9087	2.06E-03	5.42E-02	High Diversity
REACTOME_TRANSCRIPTIONAL_REGULATION_BY_E2F6	6/364	34/9087	2.06E-03	5.42E-02	High Diversity
PID_ERBB1_RECEPTOR_PROXIMAL_PATHWAY	6/364	35/9087	2.40E-03	5.49E-02	High Diversity
PID_FAS_PATHWAY	6/364	35/9087	2.40E-03	5.49E-02	High Diversity
REACTOME_SIGNALING_BY_FGFR3	6/364	35/9087	2.40E-03	5.49E-02	High Diversity
PID_RET_PATHWAY	6/364	37/9087	3.22E-03	6.81E-02	High Diversity
REACTOME_OVARIAN_TUMOR_DOMAIN_PROTEASES	6/364	38/9087	3.69E-03	7.46E-02	High Diversity
KEGG_MEDICUS_REFERENCE_ITGA_B_FAK_RAC_SIGNALING_PATHWAY	8/473	28/9087	6.26E-05	6.00E-03	Low Diversity
REACTOME_PLASMA_LIPOPROTEIN_CLEARANCE	8/473	35/9087	3.44E-04	1.82E-02	Low Diversity
REACTOME_GAP_JUNCTION_TRAFFICKING_AND_REGULATION	8/473	39/9087	7.48E-04	2.86E-02	Low Diversity
REACTOME_ADHERENS_JUNCTIONS_INTERACTIONS	8/473	44/9087	1.72E-03	4.83E-02	Low Diversity
KEGG_MEDICUS_REFERENCE_ITGA_B_TALIN_VINCULIN_SIGNALING_PATHWAY	9/473	28/9087	7.39E-06	1.01E-03	Low Diversity
PID_A6B1_A6B4_INTEGRIN_PATHWAY	9/473	44/9087	3.60E-04	1.82E-02	Low Diversity
REACTOME_EPH_EPHRIN_MEDIATED_REPULSION_OF_CELLS	9/473	48/9087	7.08E-04	2.83E-02	Low Diversity
REACTOME_ASSEMBLY_OF_COLLAGEN_FIBRILS_AND_OTHER_MULTIMERIC_STRUCTURES	9/473	52/9087	1.29E-03	4.12E-02	Low Diversity
PID_HIF1_TFPATHWAY	9/473	57/9087	2.51E-03	5.44E-02	Low Diversity
REACTOME_SIGNALING_BY_FGFR1_IN_DISEASE	7/364	33/9087	2.71E-04	3.36E-02	High Diversity
REACTOME_IRS_MEDIATED_SIGNALLING	7/364	37/9087	5.68E-04	4.54E-02	High Diversity
PID_TCPTP_PATHWAY	7/364	39/9087	7.92E-04	4.54E-02	High Diversity
REACTOME_SIGNALING_BY_TYPE_1_INSULIN_LIKE_GROWTH_FACTOR_1_RECEPTOR_IGF1R	7/364	41/9087	1.08E-03	4.54E-02	High Diversity
REACTOME_INSULIN_RECEPTOR_SIGNALLING_CASCADE	7/364	42/9087	1.25E-03	4.54E-02	High Diversity
PID_FOXO_PATHWAY	7/364	46/9087	2.17E-03	5.49E-02	High Diversity
REACTOME_SYNTHESIS_OF_PIPS_AT_THE_PLASMA_MEMBRANE	7/364	49/9087	3.14E-03	6.81E-02	High Diversity
PID_FCER1_PATHWAY	7/364	51/9087	3.95E-03	7.47E-02	High Diversity
REACTOME_SIGNALING_BY_FGFR_IN_DISEASE	7/364	54/9087	5.46E-03	9.68E-02	High Diversity
REACTOME_BACTERIAL_INFECTION_PATHWAYS	10/473	66/9087	2.02E-03	4.97E-02	Low Diversity
PID_MYC_ACTIV_PATHWAY	10/473	76/9087	5.77E-03	9.70E-02	Low Diversity
REACTOME_GLUCOSE_METABOLISM	10/473	76/9087	5.77E-03	9.70E-02	Low Diversity
PID_ERBB1_INTERNALIZATION_PATHWAY	8/364	40/9087	1.53E-04	2.22E-02	High Diversity
REACTOME_REGULATION_OF_CHOLESTEROL_BIOSYNTHESIS_BY_SREBP_SREBF	8/364	55/9087	1.44E-03	4.99E-02	High Diversity
REACTOME_PLASMA_LIPOPROTEIN_ASSEMBLY_REMODELING_AND_CLEARANCE	11/473	58/9087	1.66E-04	1.25E-02	Low Diversity
PID_LYSOPHOSPHOLIPID_PATHWAY	11/473	59/9087	1.95E-04	1.25E-02	Low Diversity

REACTOME_RHOC_GTPASE_CYCLE	11/473	74/9087	1.44E-03	4.45E-02	Low Diversity
REACTOME_RAC2_GTPASE_CYCLE	11/473	87/9087	5.26E-03	9.51E-02	Low Diversity
REACTOME_HEME_SIGNALING	9/364	45/9087	5.93E-05	1.29E-02	High Diversity
REACTOME_RNA_POLYMERASE_II_TRANSCRIPTION_TERMINATION	9/364	66/9087	1.18E-03	4.54E-02	High Diversity
REACTOME_NON_INTEGRIN_MEMBRANE_ECM_INTERACTIONS	12/473	52/9087	1.06E-05	1.26E-03	Low Diversity
REACTOME_SIGNALING_BY_MET	12/473	74/9087	3.95E-04	1.89E-02	Low Diversity
REACTOME_COLLAGEN_FORMATION	12/473	78/9087	6.48E-04	2.82E-02	Low Diversity
REACTOME_PI_METABOLISM	10/364	77/9087	9.40E-04	4.54E-02	High Diversity
REACTOME_POTENTIAL_THERAPEUTICS_FOR_SARS	10/364	85/9087	2.02E-03	5.42E-02	High Diversity
HALLMARK_PI3K_AKT_MTOR_SIGNALING	13/473	94/9087	1.10E-03	3.77E-02	Low Diversity
PID_CXCR4_PATHWAY	14/473	82/9087	7.44E-05	6.48E-03	Low Diversity
REACTOME_REGULATION_OF_INSULIN_LIKE_GROWTH_FACTOR_IGF_TRANSPORT_AND_UPTAKE_BY_INSULIN_LIKE_GROWTH_FACTOR_BINDING_PROTEINS_IGFBPS	14/473	89/9087	1.85E-04	1.25E-02	Low Diversity
REACTOME_DEGRADATION_OF_THE_EXTRACELLULAR_MATRIX	14/473	102/9087	7.75E-04	2.86E-02	Low Diversity
PID_P53_REGULATION_PATHWAY	11/364	59/9087	1.82E-05	5.26E-03	High Diversity
REACTOME_TCR_SIGNALING	11/364	99/9087	1.96E-03	5.42E-02	High Diversity
REACTOME_SIGNALING_BY_VEGF	15/473	100/9087	1.90E-04	1.25E-02	Low Diversity
REACTOME_CIRCADIAN_CLOCK	12/364	68/9087	1.36E-05	5.26E-03	High Diversity
PID_ERBB1_DOWNSTREAM_PATHWAY	12/364	104/9087	8.86E-04	4.54E-02	High Diversity
REACTOME_RESPONSE_TO_ELEVATED_PLATELET_CYTOSOLIC_CA2	16/473	101/9087	5.97E-05	6.00E-03	Low Diversity
REACTOME_FC_EPSILON_RECEPTOR_FCERI_SIGNALING	13/364	122/9087	1.16E-03	4.54E-02	High Diversity
REACTOME_CDC42_GTPASE_CYCLE	17/473	150/9087	2.00E-03	4.97E-02	Low Diversity
HALLMARK_XENOBIOTIC_METABOLISM	17/473	159/9087	3.70E-03	7.09E-02	Low Diversity
HALLMARK_ESTROGEN_RESPONSE_LATE	19/473	179/9087	2.41E-03	5.44E-02	Low Diversity
HALLMARK_HYPOXIA	19/473	187/9087	3.93E-03	7.38E-02	Low Diversity
REACTOME_FORMATION_OF_THE_CORNIFIED_ENVELOPE	20/473	95/9087	6.42E-08	2.05E-05	Low Diversity
REACTOME_KERATINIZATION	20/473	100/9087	1.58E-07	3.02E-05	Low Diversity
HALLMARK_EPITHELIAL_MESENCHYMAL_TRANSITION	20/473	187/9087	1.71E-03	4.83E-02	Low Diversity
HALLMARK_MITOTIC_SPINDLE	20/473	199/9087	3.55E-03	6.94E-02	Low Diversity
REACTOME_CELL_JUNCTION_ORGANIZATION	21/473	97/9087	1.78E-08	8.52E-06	Low Diversity
HALLMARK_GLYCOLYSIS	22/473	187/9087	2.75E-04	1.55E-02	Low Diversity
REACTOME_CELL_CELL_COMMUNICATION	23/473	128/9087	1.48E-07	3.02E-05	Low Diversity
HALLMARK_P53_PATHWAY	23/473	199/9087	2.62E-04	1.55E-02	Low Diversity
REACTOME_METABOLISM_OF_CARBOHYDRATES	26/473	252/9087	6.40E-04	2.82E-02	Low Diversity
REACTOME_PLATELET_ACTIVATION_SIGNALING_AND_AGGREGATION	27/473	204/9087	6.70E-06	1.01E-03	Low Diversity
REACTOME_MRNA_SPLICING	21/364	212/9087	1.14E-04	1.98E-02	High Diversity

REACTOME_NEDDYLATION	21/364	230/9087	3.55E-04	3.55E-02	High Diversity
HALLMARK_APICAL_JUNCTION	30/473	182/9087	1.49E-08	8.52E-06	Low Diversity
REACTOME_PROCESSING_OF_CAPPED_INTRON_CONTAINING_PRE_MRNA	29/364	280/9087	2.36E-06	2.05E-03	High Diversity
